# Towards large-scale programmable silicon photonic chip for signal processing

**DOI:** 10.1515/nanoph-2023-0836

**Published:** 2024-02-19

**Authors:** Yiwei Xie, Jiachen Wu, Shihan Hong, Cong Wang, Shujun Liu, Huan Li, Xinyan Ju, Xiyuan Ke, Dajian Liu, Daoxin Dai

**Affiliations:** State Key Laboratory for Modern Optical Instrumentation, Center for Optical & Electromagnetic Research, College of Optical Science and Engineering, International Research Center for Advanced Photonics, Zhejiang University, Hangzhou 310058, China; Centre for Optical and Electromagnetic Research, State Key Laboratory for Modern Optical Instrumentation, International Research Center for Advanced Photonics (Hanining), Zhejiang University, Hangzhou 310058, China; Advance Laser Technology Laboratory of Anhui Province, Hefei 230037, China; Ningbo Research Institute, Zhejiang University, Ningbo 315100, China

**Keywords:** large-scale, programmable optical signal processing, ultra-low-loss, silicon photonic waveguides, low phase-error, switch

## Abstract

Optical signal processing has been playing a crucial part as powerful engine for various information systems in the practical applications. In particular, achieving large-scale programmable chips for signal processing are highly desirable for high flexibility, low cost and powerful processing. Silicon photonics, which has been developed successfully in the past decade, provides a promising option due to its unique advantages. Here, recent progress of large-scale programmable silicon photonic chip for signal processing in microwave photonics, optical communications, optical computing, quantum photonics as well as dispersion controlling are reviewed. Particularly, we give a discussion about the realization of high-performance building-blocks, including ultra-low-loss silicon photonic waveguides, 2 × 2 Mach–Zehnder switches and microring resonator switches. The methods for configuring large-scale programmable silicon photonic chips are also discussed. The representative examples are summarized for the applications of beam steering, optical switching, optical computing, quantum photonic processing as well as optical dispersion controlling. Finally, we give an outlook for the challenges of further developing large-scale programmable silicon photonic chips.

## 1 Introduction

As Moore’s law scaling come to an end, new devices and architectures are being explored. Integrated optical signal processors are under extensive investigations to address the rapidly growing need for high bandwidth, low latency, and power efficiency in signal processing of various applications [1], [2], [3]. Assisted with tuning elements, the so-called programmable processor chips are able to switch between different functions by means of programming, and also can be trimmed and optimized post-production. This not only provides a path to significantly reducing the cost per function, but more importantly, one common verified design can be used for many applications [4], [5], [6], [7]. In practical applications, the number of devices on a single PIC keeps increasing thanks to the high device yield and the application driven requirements for large scale PICs in areas including microwave photonics [4], [5], [6], [7], high-speed transceivers [8], optical computing [9], [10], [11], [12] and quantum photonics [13]. With ever-increasing number of channels, low loss and footprint compactness are necessary to maintain the optical signal integrity in large-scale PICs [14], [15]. Among various platforms, silicon photonics has been very popular to form the backbone for recent progress in optical transceivers, LIDARs, etc. Meanwhile, it features compact sizes (due to sharp bends), strong thermo-/electro-optic effects, and CMOS compatibility [16]. Thus, silicon photonic chips become attractive candidates to realise low-cost and programmable signal processing.

To date, numerous silicon photonic processors using different mesh structures with multiport devices and arrays have been successfully demonstrated for key optical signal processing functions, including 1 × 8 microring-resonators (MRRs) delayline processor for image processing with switchable convolution kernels [17], a waveguide mesh composed of seven hexagonal cells capable of implementing over 100 different circuit layouts and functions [18], a 8 bit tunable delayline for tunable delay [19], as well as 8 × 8 Mach–Zehnder interferometer (MZI)-mesh processor for multichannel optical switching, optical descrambler, and tunable optical filter [20]. Although these programable optical processors have successfully demonstrated versatile functions, it is still very challenging to further scale up the chip for more function realizations and powerful information processing.

The first challenge is how to significantly lower the loss of silicon photonic waveguides, regarding that regular silicon photonic waveguides usually have a propagation loss of ∼2 dB/cm. In the past decades, tremendous efforts have been devoted to reduce the propagation loss and increase the chip scale [21], [22], [23], [24], [25]. The ultra-thin low-loss photonic waveguide has been used for developing a tunable optical signal processors with the maximum delay of >1 ns and an excess loss of 12.4 dB, which can be programmed to achieve optical pulse time-division multiplexing and quasi-arbitrary waveform generation. Moreover, it has extended to an 8-channel delayline array for radar and wireless communication systems [21]. With low-loss ridge silicon photonic waveguides, a higher-order microring resonator with a bandwidth of a few GHz is possible [22]. However, for these reported low-loss silicon photonic waveguides, the bending radius is usually quite large because of the weakened mode confinement, and their fabrication is not fully compatible with current multi-project-wafer (MPW) foundry processes. To address such issues, we have proposed an ultra-low-loss broadened silicon waveguides, which are far beyond the single mode regime and can be fabricated easily with standard foundry fabrication processes, showing the measured loss as low as 0.14 dB/cm [23]. The low-loss silicon photonic waveguides have further been used for realizing a 4-channel chip-scale programmable optical signal processor with on-chip loss of 3–3.4 dB [24] and the 16-channel wavelength-selective signal processors with insertion loss of 10 dB [25], which enables to perform a number of distinctive functionalities like tunable time-delay and microwave photonic beamforming

In addition, sophisticated programmable optical processors usually require hundreds or even thousands of tunable basic building blocks, such as 2 × 2 Mach–Zehnder switches (MZSs) [18], [21], MRRs [10], [25] and dispersion controllers [26]. There might be significant device performance nonuniformity and errors caused by the fabrication variations. When the fabrication inconsistency in the basic building blocks is accumulated, risks for function failure increase significantly. Improved processor architectures (including FFTNet topologies and redundant topologies [27]) and self-configuring algorithms [28], [29] are both playing important role to enhance the robustness. However, the calibration is quite complicated and many additional elements (such as tap couplers and power monitors) are needed to assist the configuration. To enhance the robustness and overcome the random fabrication errors, the most important key is to improve the fabrication tolerance of basic building blocks. Recently, it is realized more that broadened silicon photonic waveguides are very helpful for not only significantly lower the propagation losses but also greatly reducing the random phase errors for light propagation. As a result, it provides a promising approach to have immunity to fabrication errors. For example, broadened silicon photonic delaylines possibly provide the way to achieve the time delay precisely [30]; Broadened phase shifters show very low random phase errors, resulting in quasi-calibration-free configuration of optical signal processors and low power consumption for phase-error compensation [31].

Here, we give a review on recent progress of large-scale programmable silicon photonic chip for signal processing in various applications. What’s more, we emphasis the challenges and solutions for realizing and controlling the large-scale programmable chips, including the waveguide loss, fabrication tolerance, power consumption, and control methods. In the first part, high-performance building-blocks is discussed, including ultra-low-loss silicon photonic waveguides, 2 × 2 MZSs and MRR switches. Configuring methods of large-scale programmable silicon photonic chips are also discussed. Then we give a summarization for the representative examples for the applications of microwave photonics, optical switching, optical computing, quantum photonic processing as well as optical dispersion controlling. Finally, an outlook is given for the challenges of developing large-scale programmable silicon photonic chips.

## 2 Basic building blocks

### 2.1 Silicon photonic waveguides

**Figure 1:**
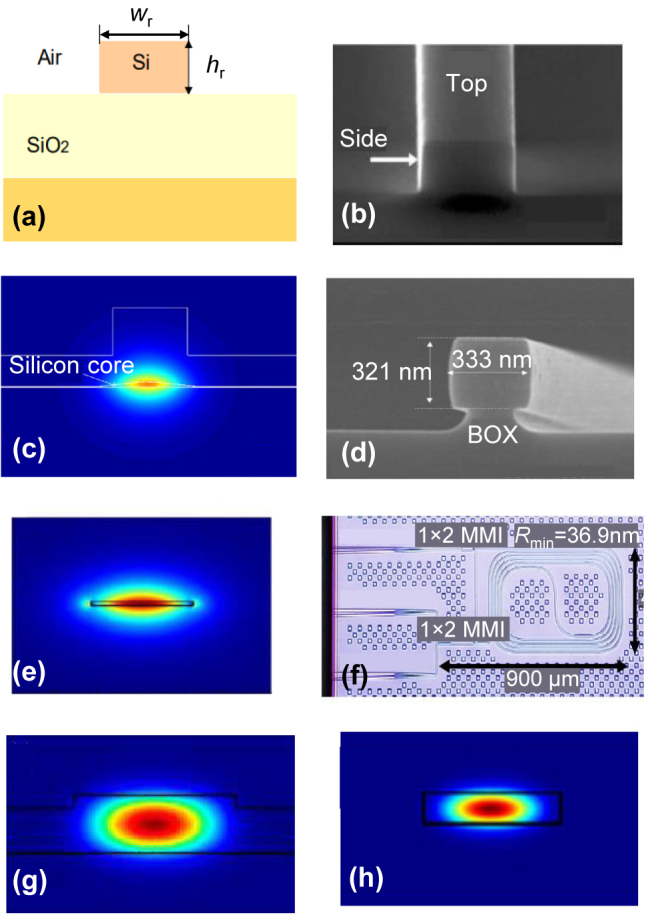
Ultra-low-loss silicon waveguide technologies. (a) The cross section of the SOI nanophotonic waveguide (0.45 μm × 220 nm); (b) picture of the traditional SOI nanophotonic waveguide. Low-loss silicon wavelength using extra fabrication methods: (c) selective oxidation without silicon etching; (d) thermal oxidation and hydrogen annealing [34]; using mode field distribution optimization using (e) ultra-thin silicon waveguide [19]; (f) thick waveguides [36]; (g) shallow-etched ridge waveguide; (h) broadened waveguide. (d) Reproduced with permission [34]. Copyright 2017, SPIE – International Society for Optics and Photonics. (e) Reproduced with permission [19]. Copyright 2017, Optica Publishing Group. (f) Reproduced with permission [36]. Copyright 2015, SPIE – International Society for Optics and Photonics.

Figure 1(a) and (b) shows the cross-section of a typical silicon-on-insulator (SOI) strip waveguide, which is a basic element for silicon photonic devices and circuits. The thickness, *h*_r_ of the top-silicon layer is chosen as tens or hundreds of nanometers, depending on the applications [32], and the width, *W*_r_ is usually set as 450 nm for singlemode transmission. In generally, it has a micro-scale bending radius and typically a propagation loss of ∼2 dB/cm, which makes it very challenging for realizing large-scale on-chip optical signal processors. For silicon photonic waveguides, the loss mainly comes from the scattering loss, the substrate leakage loss and the bending loss. Among them, the scattering losses caused by the surface roughness are the dominant. In terms of reducing the scattering loss, there are usually two approaches. One is developing new fabrication processes to smooth the waveguide sidewalls [33], [34], and the other is optimizing the mode field distribution to reduce interaction between the light field and the waveguide sidewalls [35], [36], [37].

As shown in Figure 1(c), a silicon photonic waveguide with a propagation loss of only 0.3 dB/cm at 1550 nm is realized by using the special selective oxidation process without etching silicon [33]. Alternatively, Bellegarde et al. used the processes of thermal oxidation and hydrogen annealing to smooth the etched waveguide core, and a silicon photonic waveguide with the propagation loss of ∼0.5 dB/cm was realized [34], as shown in Figure 1(d).

In contrast, a *h*_r_ = 60 nm thick ultrathin silicon photonic waveguide, as shown in Figure 1(e), was fabricated with a low loss of 0.6 dB/cm [19], [35], because less interaction of the optical field with the sidewalls for the ultra-thin silicon photonic waveguides whose sidewall area is reduced greatly compared to those regular 220-nm-thick SOI strip waveguides. One should note that significantly increasing the silicon core thickness is also an effective way to reduce the propagation loss. For example, Cherchi et al. have demonstrated low propagation losses for strip silicon photonic waveguides whose thickness is 3–4 μm [36]. As shown in Figure 1(f), a 1-cm-long delayline was fabricated and the measured loss is ∼0.16 dB/cm. Additionally, a shallowly-etched silicon photonic ridge waveguide has also been used for reducing the interaction between the model field and the sidewalls. The shallowly-etched silicon photonic ridge waveguides, whose width and height are respectively 2 µm and 0.25 µm, have been demonstrated and shown an average propagation loss of 0.27 dB/cm in the C-band [Figure 1(g)]. Here the etch depth for the ridge waveguide is 0.05 µm [37]. Nevertheless, for these silicon photonic waveguides, the bending radius usually has to be quite large because of the weak mode confinement. Furthermore, their fabrication is not fully compatible with current multiproject-wafer (MPW) foundry processes. In order to solve this problem, we have proposed ultra-low-loss broadened silicon photonic waveguides with the width, *W*_r_ increased to 1.6 μm, showing a low propagation loss of 0.28 dB/cm, as given in Figure 1(h). One should note that this silicon photonic waveguide uses the single-etching process, and no extra fabrication process is needed. In addition, they fabricated very long tunable delayline (with 0–5.12 ns) assisted by the compact Archimedean spiral waveguides with a tapered Euler S-bend [38]. Furthermore, when the waveguide width is even increased to 3 μm with waveguide loss of 0.14 dB/cm, the silicon MRR based on the ultra-low loss silicon waveguide has the record high Q-factor of 10^7^ so far [23].

As a summary, currently there are several methods to reducing the loss of silicon photonic waveguides by improving the fabrication processes for smoothing the waveguide surfaces or optimizing the field intensity at the sidewalls, as summarized in Table 1. Even though these methods using special fabrication processes are effective for reducing the propagation loss to <0.6 dB/cm [33], [34], [35], [36], they require extra fabrication processes, which increase the cost greatly and make it not easy for massive fabrication. Instead, one can simply employ the multimode waveguide using a standard fabrication process, the waveguide can be reduced to 0.14 dB/cm [23].

**Table 1:** Methods to reduce the silicon waveguide loss.

Waveguide	Fabrication processes	Cross section	Length (cm)	Loss (dB/cm)
Single mode waveguide [32]	Single etching	*w*_r_ = 0.45 μm *h*_r_ = 0.34 μm	–	2
Strip waveguide [19]	Single etching and thin silicon and oxidation	*w*_r_ = 1.00 μm *h*_r_ = 0.06 μm	8	0.61
Ridged waveguide [37]	Double etching	*w*_r_ = 2.00 μm *h*_r_ = 0.05 μm *h*_s_ = 0.20 μm	64	0.274
Irregular waveguide [33]	Oxidation and annealing	–	4	0.3
Strip waveguide [34]	Thermal oxidation and hydrogen annealing	*w*_r_ = 0.33 μm *h*_r_ = 0.32 μm	–	0.5
Strip waveguide [36]	Single etching and thick silicon	*h*_r_ = 3.0 μm	1	0.15
Strip waveguide [23]	Single etching	*w*_r_ = 3.0 μm *h*_r_ = 0.22 μm	100	0.14

### 2.2 Silicon photonic switches

The ever-growing demand for real-time processing of big data streams, fast switching and routing in transmission links poses requirements of higher performance for optical switches [39]. For optical information processing, optical switches also serve as key components for constructing large-scale optical switch arrays and are critical for the overall performance of optical networks. Compared with electrical switches, optical switches eliminate the O-E-O conversion, simplifying the device and enhancing network reliability. Although electrical switching is utilized in current communication systems, optical networks in the future aims to eliminate O-E-O conversion through optical switches, enabling the function of signal routing with more convenience of high speed, and protocol transparency. Consequently, the importance of optical switch cells and arrays is increasingly prominent. In practical, the performance requirements of on-chip optical switches primarily include low losses, high extinction ratios, low power consumption, large fabrication tolerance and low polarization sensitivity. In following section, we describe the two key optical switches for optical signal processing, including Mach–Zehnder switches (MZSes) and MRRs. The device optimizations of these two types of optical switches are illustrated, such as excess losses, robustness, power consumption, and tuning mechanisms.

#### 2.2.1 Mach–Zehnder switches (MZSes)

**Figure 2:**
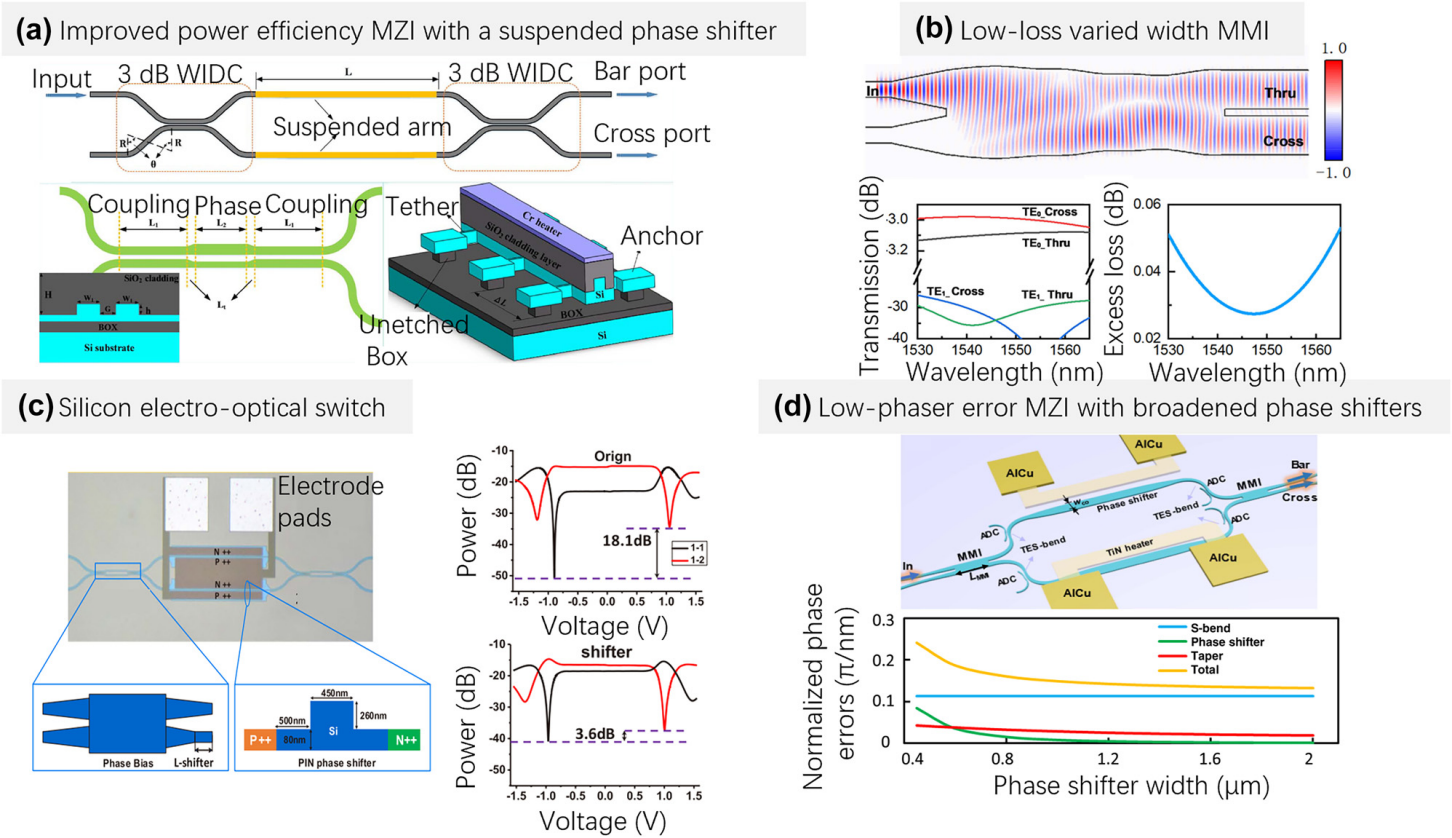
Representative MZI switch designs. (a) Schematic structure of the high-power-efficiency silicon thermo-optic switch using a suspended phase shifter [40]; (b) varied width ultra-low loss MMI with PSO algorithm [46]; (c) schematic diagram of the structure and phase bias effect of the silicon electro-optical switch [49]; (d) low phase-error MZI with broadened phase shifters [31]. (a) Reproduced with permission [40]. Copyright 2019, Optica Publishing Group. (b) Reproduced with permission [46]. Copyright 2022, IEEE. (c) Reproduced with permission [49]. Copyright 2016, Optica Publishing Group. (d) Reproduced with permission [31]. Copyright 2021, Optica Publishing Group.

Generally, MZSes working with the thermo-optic (TO) effects have been used popularly in large-scale optical signal processors, which are because these devices feature easy fabrication, low excess losses, and high extinction ratios. For TO MZSes, the power consumption is mainly determined by the heat transfer through the silica upper-cladding and the foundry fabrication processes of the heaters, which could cause serious thermal crosstalk and influence the working point. In order to reduce the power consumption, in 2019, K. Chen et al. demonstrated a TO silicon photonic switch [in Figure 2(a)], which combines a 2 × 2 3-dB coupler with a laterally-supported suspended phase-shifter arm and a highly efficient metal heater [40]. Particularly, a ridge waveguide directional coupler with phase control has also been introduced as a broadband (∼100 nm) 2 × 2 3-dB power splitter. With the suspended phase-shifter arm, heating efficiency can be dramatically increases, while the mechanical stability is maintained. The design achieves a very low switching power of 1.1 mW, and the switching power can be reduced by about 50 %, while the rise/fall time switching response times are 76/48 μs. When introducing air-trenches at the sides/bottom of the silicon core, the heating efficiency improved greatly to 2.4 mW/FSR [41], 4 mW/FSR [42], even 5 mW/FSR [43], while the response time becomes as long as 10^2^ µs. Although high-power-efficient phase shifters have been designed [41], [42], [43], mutual thermal crosstalk is still a big issue when the processor chips’ scale is increased, in order to mitigate the thermal crosstalk effect, several potential approaches could be introduced. First, optimize the chip layout to guarantee sufficient special separation between the heaters [44]; Second, introduce some special designs to isolate thermal diffusion as well as improve the thermal efficiency, such as thermal isolation trenches, suspended structures, etc. [25]; Third, develop temperature-insensitive photonic devices [45].

As for the loss of the TO MZSes, they are mainly from the 3-dB couplers, which are made up of multimode-interference couplers (MMICs) or directional couplers (DCs). The MMIC features better fabrication tolerances and compacter sizes than DCs in terms of the coupling ratio and the operation bandwidth. On the other hand, the MMI loss is generally higher than that of DC. In 2021, we proposed and designed a width-varied MMIC by employing particle swarm optimization (PSO) to [46], as shown in Figure 2(b). From this figure, it can be seen that an ultra-low loss of <0.0053 dB and a higher-order mode suppression of <−26 dB in the C-band (1530–1565 nm) can be achieved in simulation. In contrast, the measured excess loss of photonic devices fabricated in standard 180-nm foundry processes is ∼0.1 dB.

In generally, TO silicon photonic switches offer microsecond-scale switching, although with doped-silicon heater, the switching time can be improved to several μs [47]. Alternatively, electro-optic (EO) switches feature nanosecond-scale switching and negligible power consumption. These features are particularly important in the application of fast switching and routing transmission links. In generally, EO silicon photonic switches employ PIN structures and the plasma dispersion effect [48]. As a result, the faster switching speed of EO switches results in high propagation losses due to free carrier absorption in silicon, compromising their scalability. Compared with TO counterparts, the length and the loss of the EO phase-shifter arm increase significantly, leading to increased propagation loss and random phase errors. In 2016, Tang et al. proposed an optical switch that simultaneously integrates EO and TO effects and reported a 16 × 16 switch array based on this elementary device [49]. Its structure and measurement results are shown in Figure 2(c). Due to the variation of foundry processes, the phase-shifter arms for each MZI with identical designs are imbalanced. They have added a π/2-optical-phase-bias in each MZI, so that the switch is initially in an intermediate state between cross and bar. Therefore, the EO MZS arm only needs to be shifted by π/2 to realize the state switching, which reduces the additional loss caused by carrier injection. The crosstalk difference between the states of “cross” and “bar” is improved from 18.1 dB to 3.6 dB, while the power consumption in the bar state is reduced from 6.24 mW to 1.9 mW, which is promising for the development of large-scale switch arrays.

Both the EO and TO MZS mentioned above accumulate random phase errors due to size deviations during fabrication. Such random phase imbalances must be calibrated and compensated meticulously for all the 2 × 2 MZSs one by one in a large-scale N × N MZS. Therefore, a large number of additional power taps as well as power monitors are often required for all or part of the 2 × 2 MZS elements, so that the optimal electrical power for their cross and bar states can be individually determined by monitoring the corresponding tapped power. However, this inevitably introduces significant excess losses. Furthermore, it also entails additional on-chip feedback control schemes and sophisticated characterization procedures, which significantly complicates the layout design and greatly increases the chip footprint as well as the chip management complexity. Besides, it also consumes extra heating power for both cross and bar states. Therefore, the research on elementary MZSs with low random phase errors (calibration-free) is particularly important.

In 2021, an MZS with low random phase errors was proposed by widening the phase shifter to 2 μm for the first time [31]. Compared to the 0.45-μm-wide conventional singlemode phase shifter, the broadened phase shifter has much better fabrication tolerance to overcome the random phase error. The measured average random phase error decreased greatly, significantly reducing the power consumption for phase error compensation. As shown in Figure 2(d), the MZS consists of two 2 × 2 3 dB multimode interference (MMI) couplers and two symmetric arms with TiN microheaters on top. Widening the phase shifter reduces random phase errors per unit waveguide length, thereby reducing accumulated random phase errors. Additionally, the introduction of the TES-bend incorporates asymmetric directional couplers (ADCs) to filter out higher-order modes excited by the MMIs. The designed TES-bend demonstrates a phase error reduction to 0.0064π/nm, approximately 22 times better than the traditional 450 nm-wide S-bend, thereby achieving a high extinction ratio, low loss, and calibration-free MZI optical switch.

In addition to the reduction of losses, power consumption, and random phase error, polarization insensitivity is particularly important in the applications of switching as well. In 2018, a large-bandwidth polarization-insensitive switch [50] based on a 340-nm SOI platform was reported, and achieved a high extinction ratio of 25 dB for TE polarization and about 15 dB for TM polarization across the C-band. Since the thickness of typical silicon photonic waveguides is 220 nm, there is a large birefringence for guided modes [51]. Therefore, most silicon photonic switches can operate with only a single polarization, limiting their practical applications. In 2022, a polarization-insensitive thermo-optic MZS was proposed by using 220 × 220 nm^2^ square silicon photonic waveguides. However, the insufficient mode confinement leads to compromised device performances. The excess loss of both polarizations in the C-band was about 0.2–4.3 dB, while the extinction ratio is about 14 dB [51].

#### 2.2.2 Microring resonator (MRR) switches

**Figure 3:**
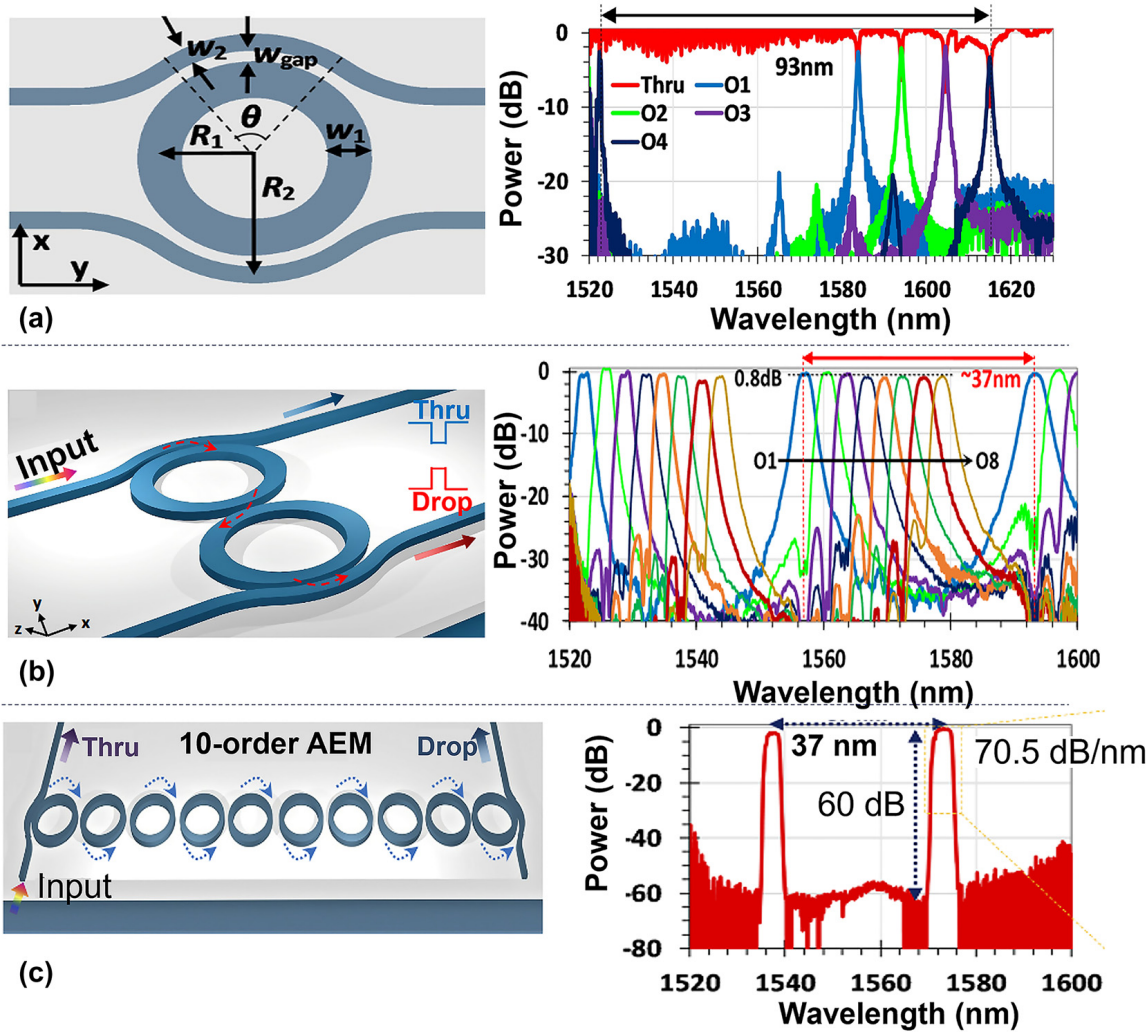
Low-loss and large-FSR MRR switch designs. (a) Schematic configuration of the silicon photonic filter based on an MRR with bent directional couplers (DCs) and the corresponding spectral responses of the four-channel add-drop filters [52]; (b) schematic configurations of the proposed two-order AEMs and its measured spectral responses at the drop ports [53]; (c) schematic configurations of the proposed ten-order AEMs and its measured spectral responses at the drop ports [54]. (a) Reproduced with permission [52]. Copyright 2019, Optica Publishing Group. (b) Reproduced with permission [53]. Copyright 2021, IEEE. (c) Reproduced with permission [54]. Copyright 2022, AIP Publishing.

MRRs have attracted lots of attention in the past decades due to its compact footprint and functional versatility. In large-scale programmable silicon photonic systems for signal processing, MRRs can be applied as wavelength-selective switches by utilizing the wavelength selectivity and the weight management. It is clear that large free spectral range (FSR), low excess losses and high extinction ratios are necessary for MRRs-based optical switches used in large-scale optical signal processors. Usually, MRRs reported previously have radii of 5–10 μm as a trade-off between the Q-factor and the FSR. The corresponding FSR is usually 10–20 nm. Recently, a low-loss add-drop optical filter with a world-record FSR of ∼93 nm was demonstrated by using an 800-nm-wide microring waveguide with a submicron bending radius of *R* = 0.8 μm, as shown in Figure 3(a) [52]. In particular, the 800-nm-wide multimode microring waveguide was introduced to achieve low-loss propagation even with a submicron bending radius (e.g., *R* = 0.8 μm). Furthermore, bent DCs were used to achieve sufficient coupling between the access waveguide and the microring waveguide. Meanwhile, the resonance of higher-order modes can be depressed well by modifying the phase-matching condition in the coupling region. As it is well known, the cascaded MRRs with a higher order enable a more box-like spectral response with higher ERs and higher roll-off rates. In [53], [54], high-order adiabatic elliptical microring (AEM) resonators were proposed. For the AEMs, the waveguides in the non-coupling regions are broadened and have a minimal bending radius, while the waveguides in the coupling regions are relatively narrow and have a maximal bending radius. Figure 3(b) and (c) shows the measured spectral responses for the two-order and 10-order AEM resonators, respectively. For this design, the FSR is as large as ∼37 nm and the ER is up to ∼60 dB for the 10-order case. Using this AEMs design, 1 × 4 wavelength-selective switch (WSS) [55] and 1 × 8 WSS [56] were then realized. The excess loss of the signals at the output port is less than 1 dB, and the crosstalk from adjacent channels is less than −20 dB.

**Figure 4:**
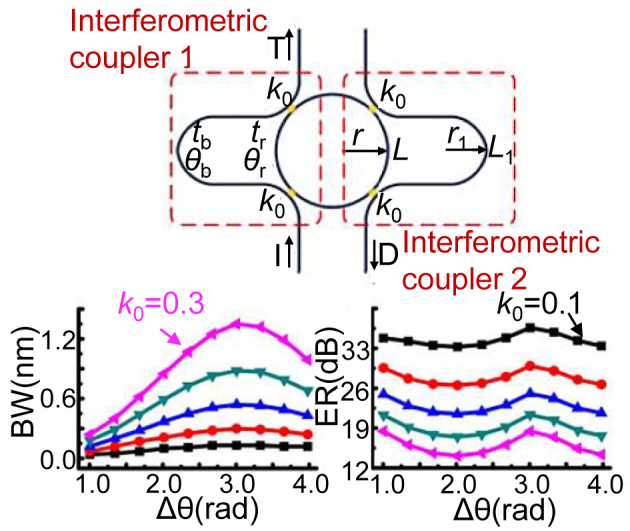
Schematic configuration of the proposed add-drop MRR with two interferometric couplers. Bandwidths (BWs) and extinction ratios (ERs) as functions of the relative phase Δ*θ* and field coupling coefficient *k*_0_ [57]. Reproduced with permission [57]. Copyright 2018, IEEE.

Usually, there is need to control both the coupling ratio and the phase delay of an MRR for the configurability of the spectral responses. When using an MRR with interferometric couplers, as shown in Figure 4 [57]. The transmission spectra of adjacent channels can be selectively combined to form adjustable bandwidths as desired. For example, the bandwidth varies from 0.12 nm to 2.91 nm, and the ER varies from 7.9 to 67 dB by choosing different coupling coefficient. Such MRRs have been applied in microwave photonic filters whose 3-dB bandwidth and central frequencies are reconfigurable [58], [59].

Table 2 shows the comparison of optical switches based on different structures. In general, MZSes [31], [40], [46], [49] have a broad operation bandwidth than MRRs, while MRRs are more compact and can be used to flexibly select the target wavelength. One should note that optical switches based on MZIs or MRRs are usually prone to random phase errors caused by the random dimensional variations introduced in fabrication processes. Consequently, these switches usually not only require extra heating power for the initialization, but also entails additional on-chip feedback control schemes with the help of many additional elements. Recently, low-phase-error MZI with broadened phase shifters have shown excellent fabrication tolerance, in which case the power consumption is reduced considerably for the phase error compensation. Such elegant methodology paves the way to further scaling up N × N silicon photonic switches and can be generalized for other phase-sensitive photonic devices as well.

**Table 2:** Performance comparison of optical switches based on MRRs and MZIs.

Ref	IL (dB)	BW (nm)	ER (dB)	Random phase error	Type of coupler	Type of switch
[31]	1	>60	∼30	0.2π	MMI	MZI
[40]	0.5	∼100	11.5	–	DC	MZI
[46]	0.35	1530–1565	∼30	0.06π	Varied-width MMI	MZI
[49]	–	1520∼1570	20.2–29.8	–	MMI	MZI
[54]	∼1	3	>60	–	Bend DC	MRR
[56]	<1	0.2	>20	–	Bend DC	MRR
[57]	<7.29	0.12–2.91	7.9–66.9	–	Interferometric couple	MRR

IL, insertion loss; ER, extinction ratio.

Other basic build blocks, such as arrayed-waveguide grating (AWG), optical crossings, are also very important in the realization of the large-scale programmable silicon photonic signal processors. With continuously improved technologies of design and fabrication, we achieved 16 × 16 AWG with a channel spacing of 1.6 nm by uniformly broadening the arrayed waveguides to be far beyond the singlemode regime. The inter-channel crosstalk at the center wavelength is as low as ∼−31.7 dB, which is ∼10 dB lower than the previously best silicon AWGs [60]. As one of the most important basic elements, optical crossings have been studied for many years. Currently it is possible to achieve optical crossings with ultra-low losses of <20 dB m by optimizing the structures [61], or introducing four identical tapered arms defined by the spline interpolation with optimized physics structures [62].

## 3 Programming strategies

When programming large-scale optical signal processors, it is usually required to control/configure hundreds of variables and simultaneously manage multiple configuration actions. Note that most reported photonic signal processors were reconfigured manually, which however is cumbersome, time-consuming, and becomes extremely difficult when the network is expanded to be very large-scale. Alternatively, self-configuring methods were proposed.

**Figure 5:**
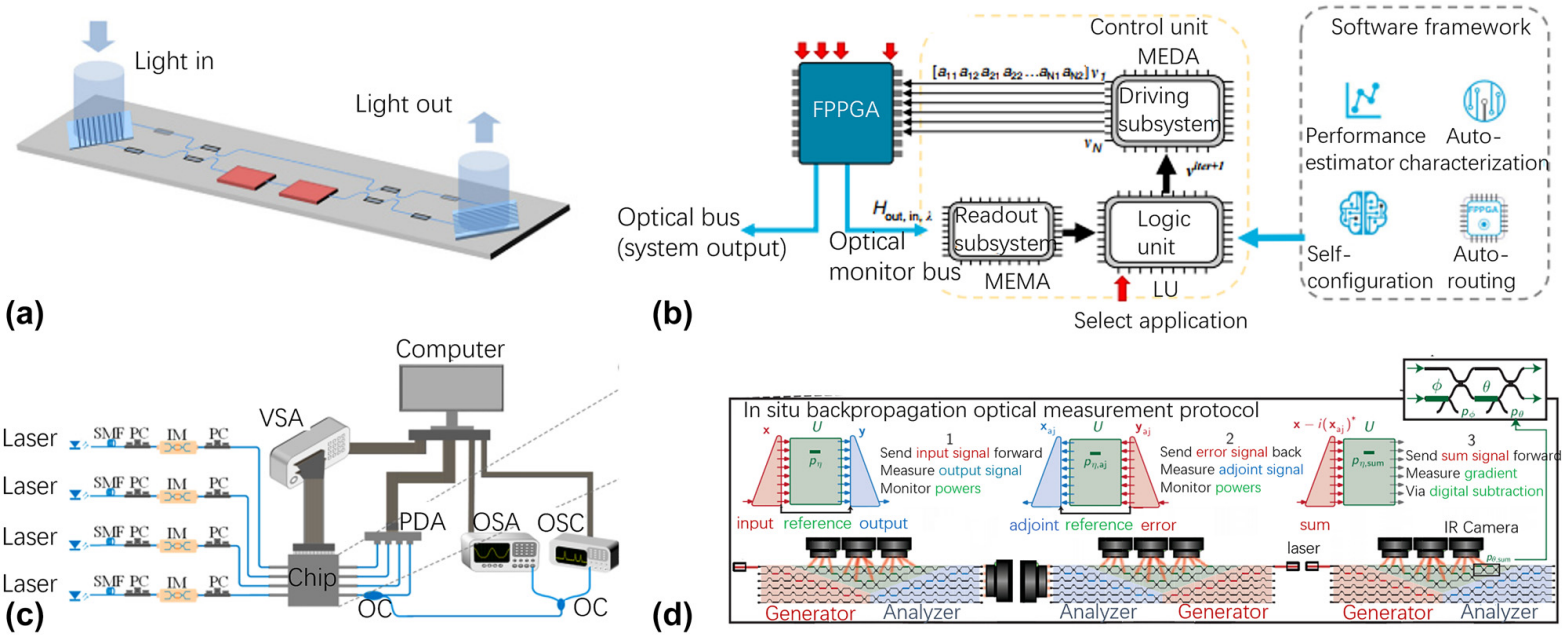
Self programming strategies using differnt methods. (a) On-chip configuring method using the built-in optical power monitors [64]; (b) self-configuring method using a logic unit processor control the readout subsystem and driving subsystem [29]; (c) self-configuring method using gradient descent algorithm for configuring multiple input and multiple output signal processors [20]. (d) “*In situ* backpropagation” method for configuring silicon photonic neural networks [65]. (a) Reproduced with permission [64]. Copyright 2013, Optica Publishing Group. (b) Reproduced with permission [29]. Copyright 2020, Nature Publishing Group. (c) Reproduced with permission [20]. Copyright 2020, American Chemical Society. (d) Reproduced with permission [65]. Copyright 2023, AAAS(American Association for the Advancement of Science).

Miller has proposed an approach using feedback from the built-in optical power monitors to configure the on-chip processors [63], [64], as shown in Figure 5(a). The entire mesh can be both optimized and programmed after the initial fabrication without calculations or calibration. In addition, Miller has also proposed an optical device which can perform any linear function or coupling between inputs/outputs using sets of detectors and local feedback loops [64]. This design method is progressive, requiring no global optimization. The chip can be self-configured progressively, avoiding the design calculations and allowing the device to stabilize itself against drifts in component properties and to continually adjust itself to changing conditions.

More automatic implementation of reconfigurable photonic signal processors is proposed to be capable of full self-configuration even without knowing the detailed information about the on-chip inner structure. It means only the input/output signals can be used for feedback, and the chip should self-learn to find the optimal solution starting from infancy. Self-configuring process [28], [29], as shown in Figure 5(b), includes logic units to connect the driving subsystem and the readout subsystem with different algorithms, such as genetic algorithm (GA), a particle swarm optimization (PSO), and gradient descent with momentum. Here GA, also known as the evolutionary algorithm, resembles natural selection/reproduction processes governed by rules that assure the survival of the fittest individuals in large populations. PSO is another population-based algorithm that maintains a swarm of particles (set of points) with a velocity vector associated with each particle for any iteration. For each iteration, a new set of particles are generated from the previous swarm combining random/inherited parameters (inertia, cognition, and social). It is typically classified as a global-search algorithm. These algorithms have been compared to configure a 36 TB hexagonal waveguide mesh for various applications [29]. With 100-times self-configuration routine, an average error of better than 3-dB in the 95 % and 76.66 % for the cases with GA and PSO, respectively (at 3000 and 4000 operations). As one of the most popular algorithms, gradient descent (GD) is an iterative first-order optimisation algorithm (see Figure 5(c)), which was presented to configure the proposed multiple-input and multiple-output photonic signal processor for performing various functions, including multichannel optical switching, optical multiple-input-multiple-output descramblers, and tunable optical filters [20].

In contrast to traditional self-configuring algorithms, which rely on numerical or automatic differentiation executed with standard computing resources, more energy-efficient “*in situ* backpropagation” algorithm has been proposed to utilize interferometric measurements to gauge the backpropagation gradient of the phase shifter voltage [65]. A photonic neural network was presented by introducing MZI-mesh networks interleaved with nonlinearities, as shown in Figure 5(d). They measured backpropagated gradients for the phase-shifter voltages by interfering forward- and backward-propagating light and simulated *in situ* backpropagation for 64-port photonic neural networks trained on MNIST image recognition given errors.

What’s more, an efficient method to configure the programmable optical processors is using low-phase-error tuning elements, as demonstrated in Section 2.2. For example, in [24], the programmable optical signal processor has 27 tunable low-phase-error MZSes, each tuning element are nearly calibration free, the voltages on all MZSs for achieving the same splitting ratio are almost the same. Such property can help reduce the calibration complexity and the power consumption greatly, particularly for a system with a large number of MZS elements.

## 4 Programmable large-scale optical signal processors

### 4.1 Large-scale delay line arrays for signal processing

**Figure 6:**
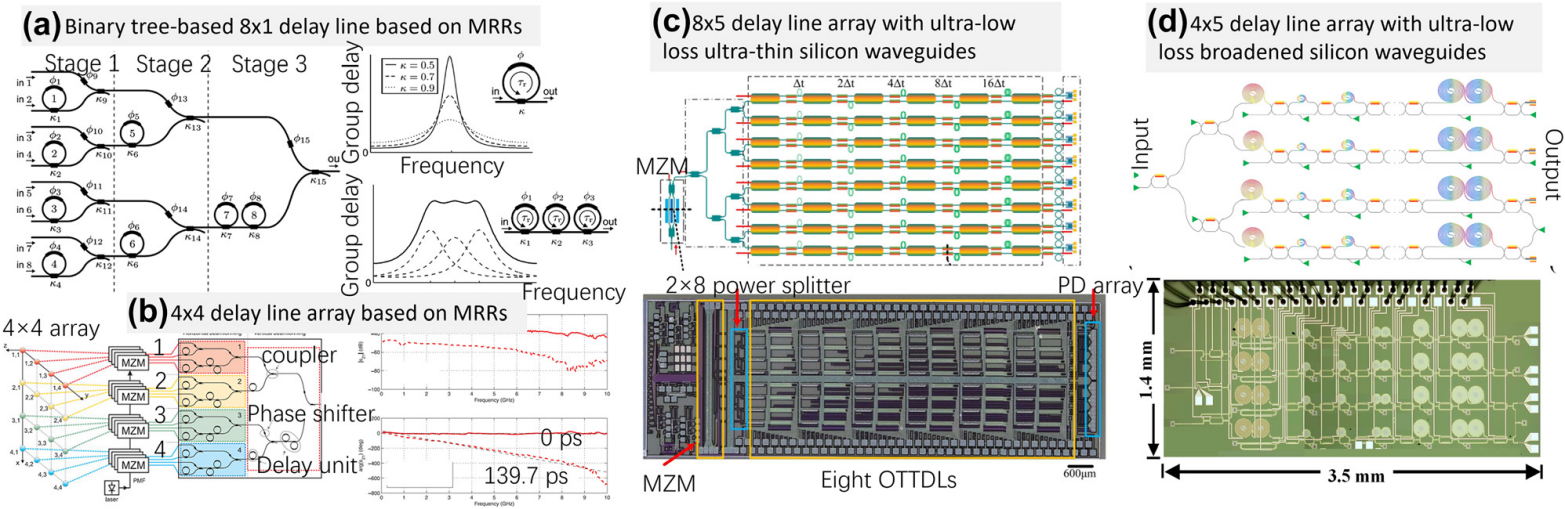
Large-scale delay line arrays using MRRs or delay spirals. (a) Binary tree-based 8 × 1 optical beam former network consisting of eight optical MRRs and seven combiners. Theoretical group delay response of the single and three cascaded ORR-based delay [66]. (b) 4 × 4 array with separable illumination and corresponding beamformer and corresponding RF magnitude and phase response of channel 1 compared to the response of channel 1 delayed [71]. (c) A phased array antenna based on an 8 × 5 optical delay line [21]. (d) 4 × 5 Low-loss chip-scale programmable silicon photonic processor with ultra-low-loss silicon waveguide and low-phase-error MZIs [24]. (a) Reproduced with permission [66]. Copyright 2010, IEEE (b) Reproduced with permission [71]. Copyright 2014, IEEE (c) Reproduced with permission [21]. Copyright 2020, Optica Publishing Group. (d) Reproduced with permission [24]. Copyright 2023, OE Journals Group.

By now, the programmable delayline systems are realized by the ways of using MRRs or long-waveguide in cascade. Figure 6(a) shows the typical optical beam former network with MRRs [66], which works at the on-resonant wavelength to provide large range and continuously-tunable delay by controlling the power coupling coefficient and the round-trip phase shift [67]. The bandwidth of an MRR-based delay element can be enhanced with the cascading configuration, as shown in Figure 6(a). A continuously tunable optical delayline with low delay ripple based on four cascaded tunable silicon-nitride MRRs for Ka-band beamforming network application was proposed in [68]. Instead of using the on-resonant wavelength to achieve the continuously-tunable delay, another option is using the anti-resonant wavelengths of MRRs as proposed in [69], [70], which leads to a large delay bandwidth and a small delay ripple. Later, a 2-D phased array antenna was proposed in [71] by employing the frequency-periodic responses of MRRs in conjunction with on-chip wavelength-division multiplexing (WDM), as shown in Figure 6(b), which creates multiple signal paths on an individual beamformer channel. This dramatically reduces the network complexity and the footprint (e.g., 36 × 8 mm^2^ [71]).

Alternatively, long-waveguide-based delayline usually consists of optical switches and multi-stages of long-waveguide. By tuning the optical switch, the delayline can be programmed digitally with a delay step in a broad wavelength range. The reconfigurable delayline on silicon [72] was proposed with the maximum of 1.27 ns and a propagation loss of 0.9 dB/cm. Furthermore, a continuously-tunable and loss-low optical delayline was reported by introducing 60-nm-thick silicon photonic waveguides with a low propagation loss of 0.61 dB/cm [19]. A 1 × 8 microwave photonic beamformer with on-chip modulators, switchable delaylines and germanium photodetectors was presented [21], as shown in Figure 6(c). It has a footprint of 42.8 mm^2^ and a delay range of 0–496 ps, a loss of ∼1.3 dB/cm and maximum power consumption of 1450 mW. This chip can support a large microwave bandwidth (8–18 GHz) without observable beam divergence and the beam angle can be tuned from 75.51° to 75.64° with an average step of 5° at 16 GHz. More recently, ultra-low-loss silicon photonic waveguides (0.28 dB/cm) were realized by introducing the structure design beyond the singlemode regime [30]. As a result, a 10 bit tunable optical delayline was demonstrated with a footprint as small as 2.2 mm × 5.9 mm and a dynamic range of 0–5120 ps. A programmable silicon photonic processor was then proposed and demonstrated [24] for the first time by introducing low-loss broaden silicon photonic waveguide spirals and low-random-phase-error MZSes, as shown in Figure 6(d). Without careful calibration of the components, this programmable silicon photonic processor can be programmed to achieve a number of distinctively different functionalities, including tunable time-delay, microwave photonic beamforming, arbitrary optical signal filtering, and arbitrary waveform generation.

### 4.2 Large-scale optical switch arrays

Large-scale optical switch arrays often play a key role for programmable optical signal processors, showing their great potential in microwave photonics, frequency measurement, microwave signal matrix calculation, and so on.

**Figure 7:**
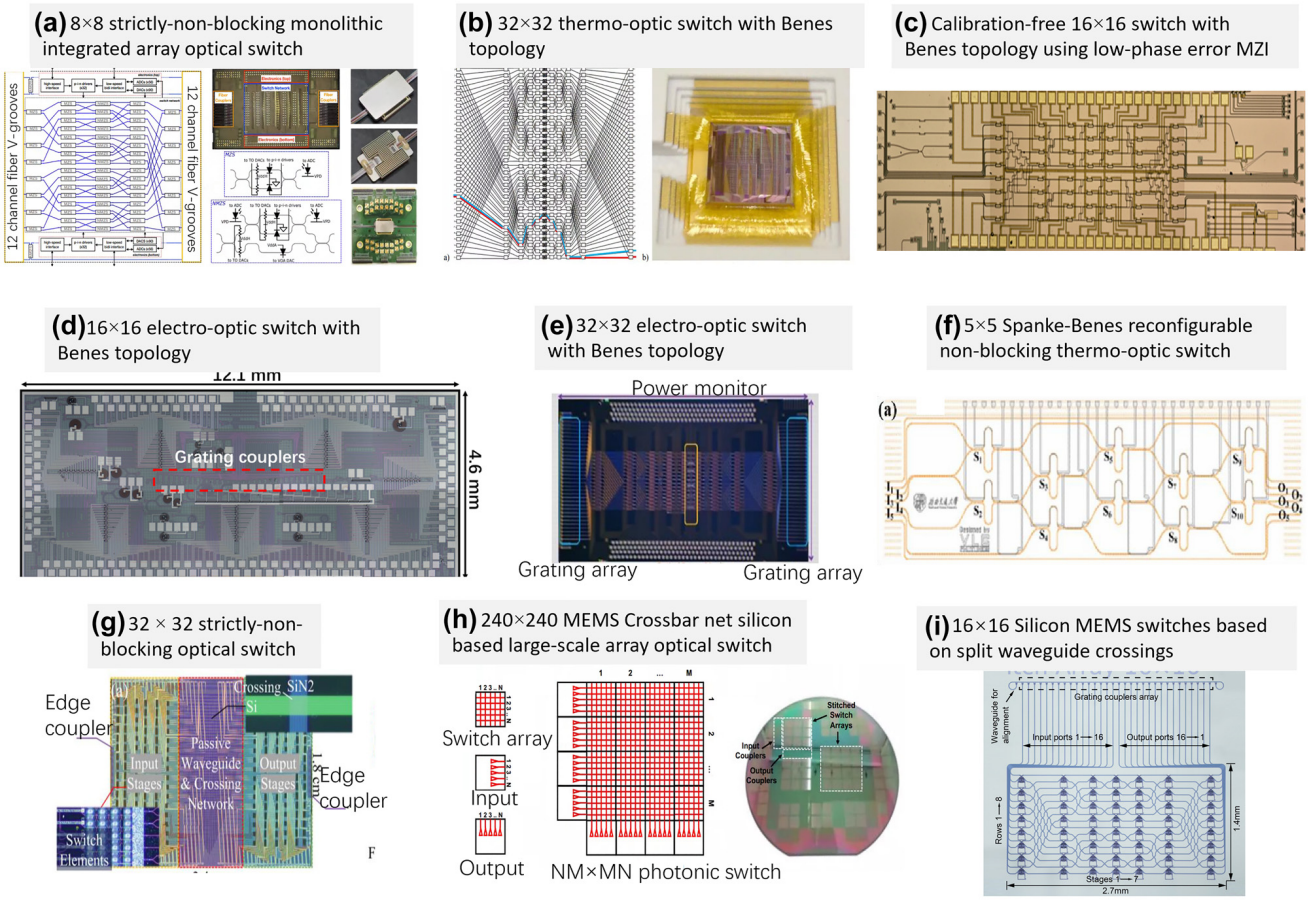
Large-scale optical switch arrays using different tuning mechanisms. (a) 8 × 8 strictly-non-blocking monolithic integrated array optical switch from IBM [73]; (b) 32 × 32 Benes topology and the physical chip packaging [74]; (c) calibration-free 16 × 16 switch with Benes topology using low-phase error MZI [75]; (d) 16 × 16 electro-optic switch with Benes topology [76]; (e) 32 × 32 electro-optic switch with Benes topology [77]. (f) 5 × 5 Spanke–Benes non-blocking thermo-optic switch [78]. (g) 32 × 32 strictly-non-blocking optical switch based on the multilayer Si3N4-on-SOI platform [79]; (h) 240 × 240 MEMS crossbar net silicon based large-scale array optical switch [80]; (i) 16 × 16 compact silicon photonic MEMS switches based on split waveguide crossings [81]. (a) Reproduced with permission [73]. Copyright 2020, IEEE. (b) Reproduced with permission [74]. Copyright 2016, IEEE. (d) Reproduced with permission [76]. Copyright 2016, Optica Publishing Group. (e) Reproduced with permission [77]. Copyright 2017, Nature Publishing Group. (f) Reproduced with permission [78]. Copyright 2019, Optica Publishing Group. (g) Reproduced with permission [79]. Copyright 2023, Wiley-VCH Verlag. (h) Reproduced with permission [80]. Copyright 2019, Optica Publishing Group.

Among various mechanisms, thermos-optic (TO) switches have been used popularly in large-scale optical switches array because of easy fabrication and low cost. Figure 7(a) shows an 8 × 8 monolithic integrated optical switch proposed and demonstrated by IBM [73], and each elementary switch has a loss of ∼0.8 dB and a switching time of ∼10 ns. It is the first time to realize a monolithically integrated optical switch with the scale of 8 × 8 and it has the very low crosstalk of <−30 dB and reasonable fiber-to-fiber coupling loss of ∼7.5–10.5 dB. Figure 7(b) demonstrates a 32 × 32 TO switch based on Benes topology [74], which consists of 448 MZI structures and 864 photodetectors. Particularly, four layers of PCBs were used to bond 1560 gold wires. For a single MZS, the switching time is ∼750 μs, the extinction ratio is ∼35 dB and the loss is ∼0.58 dB, while the on-chip loss of the array is about 23–28 dB. However, the control of such large-scale programmable optical switch array requires great effort. More recently, a calibration-free 16 × 16 TO switch was demonstrated with the Benes topology [75], as shown in Figure 7(c), in which broadened MZI arm waveguides with tapered Euler S-bends are employed to suppress the random phase imbalance for each MZS. Even without any calibration or post-compensation for the random phase imbalance of the elementary MZSs, the switch array features low crosstalk <−22 dB across the C band for the “all-cross” state. This is very helpful for achieving larger-scale optical switches in the future.

Switching speed is also an important character for large-scale switch array. In generally, EO switch has faster switching than TO switches. Figure 7(d) shows a 16 × 16 EO switch based on Benes topology [76]. Where the PIN junctions are used for realizing fast switching with ∼3 ns. The elementary switch is designed by using an MZI with dual microrings, which is helpful to realize the phase shift of π with low power consumption. Here the power consumption of each optical switch is ∼0.34 mW, the excess loss of the whole array is ∼10.6 dB. Figure 7(e) shows the 32 × 32 EO switch [77] which has a loss of 12.9–18.5 dB, an extinction ratio of 15–24.8 dB and a switching time of 1 ns.

To reduce the overall chip loss, silicon nitride has been used to fabricate low-loss optical switches. Figure 7(f) shows a 5 × 5 Spanke–Benes reconfigurable non-blocking TO switch based on silicon nitride [78]. The switching speed of millisecond, and the extinction ratio is >25 dB across a bandwidth of 80 nm (1520–1600 nm). Figure 7(g) shows a 2 × 32 switch-and-select optical switch based on silicon nitride waveguides [79]. This array contains 1984 switch units based on broadband TO MZSs, 246016 three-dimensional (3D) waveguide crossings and 2048 interlayer coupler. The loss of the 3D waveguide crossings is ∼1 mdB and the loss of an interlayer coupler is ∼0.3 dB, which significantly reduce the total excess loss and the footprint of the entire switch array. The average fiber-to-fiber insertion loss at the wavelength of 1580 nm is 12.88 dB, and the crosstalk is <−20.7 dB within the bandwidth of 110 nm, while the power consumption of the chip is only 0.98 W. The high-fidelity optical transmission of 50 Gb/s orthogonal-phase-shift-keying signals verifies the high-performance routing capability of this SiN optical switch array, which is promising for the applications in datacenter optical networks due to the broad bandwidth, the low crosstalk, and high-power efficiency.

Besides, MEMS switches feature high extinction ratios and negligible static power consumption. They have also been widely investigated for large-scale optical switch arrays. Figure 7(h) shows a 240 × 240 silicon photonic MEMS switch proposed and demonstrated recently with high extinction ratios of >70 dB, low excess loss of ∼9.8 dB, fast switching time of 0.4 μs and broad bandwidth of ∼100 nm [80], which provides the largest scale for photonic MEMS switches with high performances. The wafer-scale cross-bar MEMS switch employs an additional amorphous silicon layer on top of the SOI, entailing additional fabrication processes and reticle stitching. More recently, a new type of compact silicon photonic MEMS switches based on split waveguide crossings was demonstrated [81], which does not require additional amorphous silicon layer and hence is compatible with foundry processes, as shown in Figure 7(i). In this case, the excess loss is 0.15–0.52/0.42–0.66 dB and the extinction ratio is >45.5/25 dB over the bandwidth of 1525–1605 nm (limited by the input/output grating couplers) for the OFF/ON states. A 16 × 16 switch array using Benes topology has also been fabricated and characterized as a proof of concept [83].

### 4.3 Programmable feedforward/backward optical signal processors

**Figure 8:**
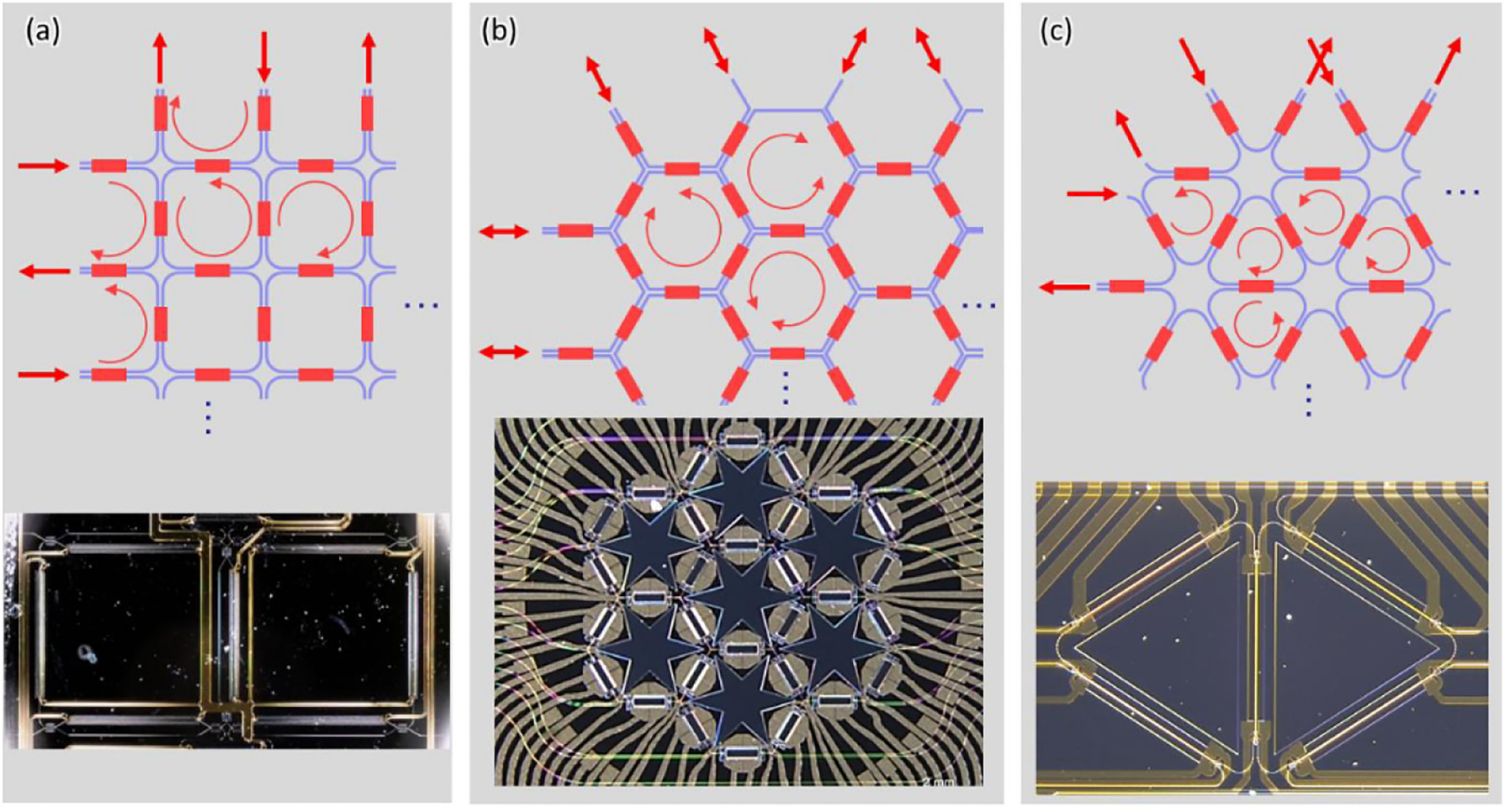
Recirculating waveguide meshes. These can be based on (a) square cells, (b) hexagonal cells and (c) triangular cells.

Programmable optical computing processors using feedforward/backward meshes offer more functional flexibility and convenient incorporation of tuning elements for microwave signal processing. In general, mesh-based computing processors use the topologies of multiport interferometers, and allow the independent amplitude and phase control of the photonic signals coupled between the two waveguides, so that it can be configured as optical networks that perform arbitrary unitary transformations on input vectors of coherent light modes [28], [82]. In 2015, for the first time, a software-defined reconfigurable microwave photonics signal processor architecture that can be integrated on a chip is proposed [83]. Then two-dimensional mesh networks consisting of tunable Mach–Zehnder couplers are proposed and analyzed. The processor can be programmed into a variety of circuit topologies to provide a variety of functions. Among programmable processors that implement reconfigurable optical cores, hexagonal mesh is the most appropriate choice of the three options (square, hexagon, and triangle) [84]. The architectures of each mesh topologies are shown in Figure 8, respectively.

These processors can be configured to achieve linear functions by interfering signals along different paths and define programmable wavelength filters, crucial for communications, and sensors. Scaling up such meshes enables linear optical computation, such as real-time matrix-vector products, which are vital in quantum information processing, neuromorphic computing, and artificial intelligence.

The key building blocks, the tunable 2 × 2 coupler and phase shifter, require low optical insertion loss and electrical power consumption. Today, most programmable photonic meshes use thermo-optic phase shifters with several mW of electrical power (in silicon). Each programmable unit cell has an insertion loss of 0.2 dB [85]. Optical waveguide losses range from 0.1–1 dB/cm depending on materials and fabrication quality. The typical example of the device is 7 hexagon meshes [28], [29]. The pioneering product from iPronics [86], the Smartlight processor, features C-band operation with 72 tuning units in a hexagonal mesh configuration and 64 input/output ports. It is capable of 50, 100 and 200 GHz bandwidth filtering functions (programmable central wavelength and extinction ratio) and demultiplexing.

### 4.4 Large-scale feedforward optical signal processors

**Figure 9:**
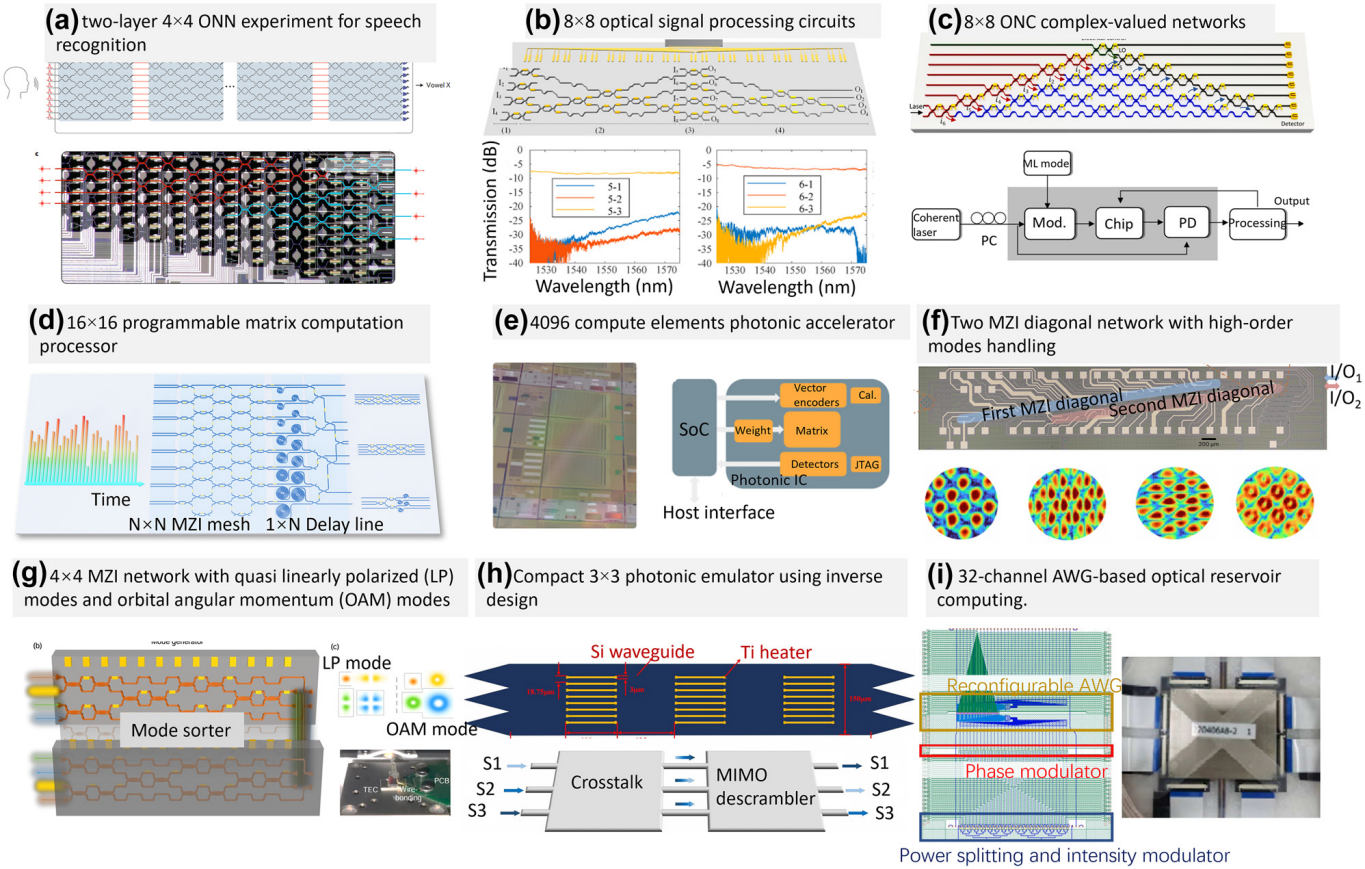
Large-scale feedforward optical signal procesors of different structures. (a) Schematic representation of two-layer 4 × 4 ONN experiment for speech recognition [9]. (b) 8 × 8 optical signal processing circuits for multichannel optical switching [20]. (c) The schematic of the 8 × 8 ONC in implementing complex-valued networks and the workflow of the system [89]. (d) 16 × 16 Programmable matrix computation processor structure for various matrix calculation [90]. (e) Micrograph of a 4096 compute elements photonic accelerator. Block diagram of the photonic accelerator including vector encoding modulators, a 4096 element PNP, and detectors [91]; (f) two MZI diagonal network with four high-order modes handling for arbitrary optical systems [92]; (g) 4 × 4 MZI network for multimode optical communication with linear polarized modes and orbital angular momentum modes [93]; (h) compact 3 × 3 photonic emulator using inverse design [94]; (i) 32-channel AWG with phase/intensity modulators as the core device to implement the on-chip optical reservoir computing [96]. (a) Reproduced with permission [9]. Copyright 2017, Nature Publishing Group. (b) Reproduced with permission [20]. Copyright 2020, American Chemical Society. (c) Reproduced with permission [89]. Copyright 2021, Nature Publishing Group. (e) Reproduced with permission [91]. Copyright 2020, Optica Publishing Group. (f) Reproduced with permission [92]. Copyright 2023, Nature Publishing Group. (g) Reproduced with permission [93]. Copyright 2023, Springer Open. (h) Reproduced with permission [94]. Copyright 2022, American Chemical Society. (i) Reproduced with permission [96]. Copyright 2023, IEEE.

Feedforward meshes using triangular interferometer [87] and improved rectangular interferometer [88] can implement any unitary transformation between a set of optical channels, this character would help to implement real/complex-valued matrix using singular value decomposition. Shen et al. firstly introduce a fully optical neural network architecture that surpasses existing electronic systems in terms of computational speed and power efficiency for conventional inference tasks [9], as shown in Figure 9(a). They validate the concept using a programmable nanophotonic processor consisting of 56 programmable Mach–Zehnder interferometers in a silicon photonic integrated circuit, showcasing its effectiveness in vowel recognition. After that, the performance and functions of the optical computing chips have been developed greatly. Dong’s group introduces a self-configuring and fully reconfigurable silicon photonic signal processor [20] (Figure 9(b)). The proposed signal processor features versatile capabilities, such as multichannel optical switching, optical multiple-input-multiple-output descrambling, and tunable optical filtering. All these functions are achieved through complete self-configuration without prior knowledge of the inner structure. Optical computing platforms can encode information in both phase and magnitude, which can be used to execute complex arithmetic by optical interference. Zhang et al. used amplitude and phase information of the optical signal, and proposed an optical neural chip that implements complex-valued neural networks [89]. Figure 9(c) shows the architecture of the 8 × 8 optical neural network, which is thermo-optically controlled to implement simple Boolean tasks, species classification of an Iris dataset, classifying nonlinear datasets (Circle and Spiral), and handwriting recognition. The processor has been scaled to 16 × 16 for parallel information processing [90], using low-loss and low-phase-error Mach–Zehnder interferometers (MZIs) for optical signal amplitude and phase manipulation. It also employs an ultralow-loss waveguide delayline array with incremental delay difference for parallel-to-serial conversion, effectively achieving signal duplication, weighting, and summation. Thanks to the device topology flexibility, the proposed processor is able to switch between a number of complex-valued matrix computations for different applications at TOPS (trillions (10^12^) of operations per second).

To meet the requirement of practical applications, a system on chip (SoC) fabricated in a 12-nm feature-size CMOS process has been proposed [91]. As shown in Figure 9(e), the SoC provides control signals to each of the 4096 photonic compute elements. In addition, the SoC contains standard electronic communication interfaces to external systems through the host interface and debugging JTAG port, as well as a large static random-access memory (RAM) cache. What’s more, in order to increase the processing information and speed, different physical dimensions of the light, such as wavelength, polarization, and mode have been investigated. In [17], a microcomb-driven chip-based photonic processing unit has been proposed to achieve convolution operation by harnessing the dimension of optical wavelength. This operation is based on time-wavelength plan stretching approach, and has been used to demonstrate image edge detection and handwritten digit recognition. Alternatively, as shown in Figure 9(f), a pair of two MZI diagonal meshes has been used to identify the orthogonal communication mode channels, corresponding to a singular value decomposition of the whole optical system [92]. Furthermore, the mode-division multiplexing system has been developed, as shown in Figure 9(g). This design uses a 4 × 4 Mach–Zehnder interferometer (MZI) mesh network, and can be configured to generate or sort four linearly polarized (LP) modes and four orbital angular momentum (OAM) modes in any desired routing state [93]. Through configuring the mesh network, it is able to switch different modes into the different output ports, and have the potential in multimode and multiwavelength communication systems. In addition, there are many novel designs to improve the performance of photonic processor in terms of device footprints and power consumption. In Figure 9(h), a photonic emulator with inverse design has been proposed by optimizing effective refractive index of the pixels in silicon photonic waveguides with 24 TiN heaters [94]. This photonic emulator has been proposed to demonstrate optical multi-input-multi-output descrambler, optical matrix computation and a tunable wavelength selective switch. Similar work was shown in ref. [12], [95], where compact on-chip diffractive optics was used and is able to control the optical power of the input signal.

In addition to optical CNN applications, reservoir computing (RC) is a kind of recurrent neural network (RNN) with a special structure, which has been implemented in integrated optical chips. Figure 9(f) shows the largest scale of the optical reservoir computing chips so far. It contains a 32-channel 50 G AWG with a TO MZI array and a phase-modulation array for information loading. The arrayed waveguide widths of the AWG are broadened to reduce the propagation loss and the random phase errors. With appropriate iterative operations and output-layer training strategy, the chip has achieved good results in predicting Macky–Glass series with a normalized root-mean-square error of 0.018 [96].

### 4.5 Large-scale integrated quantum photonics

Quantum technologies are a new type of device that can manipulat the superposition and entanglement of quantum states of light or matter in order to achieve fundamental performance benefits over traditional classical machines. The advancement of integrated quantum photonics technology now allows for the generation, processing, and detection of quantum states of light at an increasingly higher scale and complexity. This is exemplified by the development from programmable devices with a limited number of components that operate on two photons and occupy centimeter-scale footprints to devices with nearly 1000 components that operate on millimeter-scale footprints and have integrated multiphoton state generation [97], [98].

**Figure 10:**
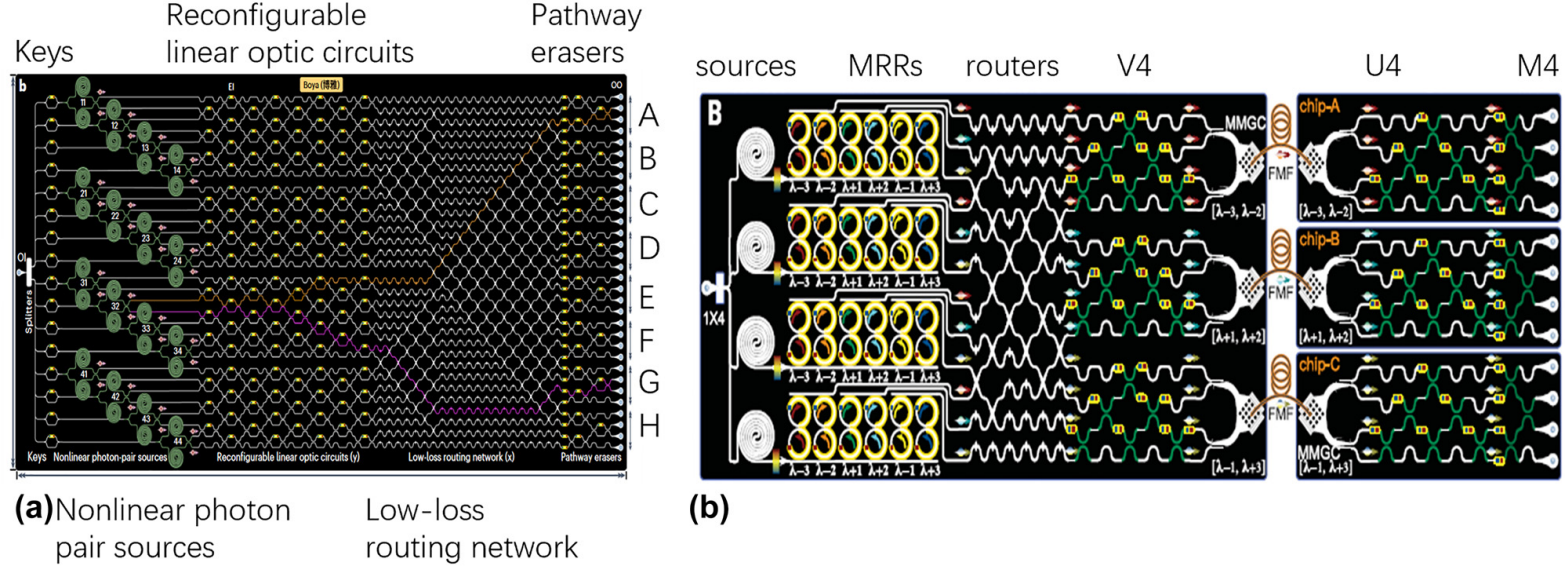
Integrated photonic devices for quantum photonics. (a) Diagrams of a graph-based quantum device with 4 × 4 nonlinear photon-pair sources in integrated optics [99]. (b) Integrated silicon-photonic quantum networking [100]. (a) Reproduced with permission [99]. Copyright 2023, Nature Publishing Group. (b) Reproduced with permission [100]. Copyright 2023, AAAS (American Association for the Advancement of Science).

As shown in Figure 10(a), very-large-scale integration (VLSI) of silicon quantum photonics was demonstrated to develop a graph-theoretical quantum photonic device with nonlinear optical sources and linear optical circuits [99]. Graph topologies may be freely reprogrammed by rearranging the device components; they are physically specified by the passage of single photons in linear optical circuits and by the connection of nonlinear optical sources. The apparatus may be used to carry out extremely broad linear optical quantum investigations.

Quantum networks serve as the foundation for quantum communication, clock synchronization, distributed quantum computation, and sensing. The creation of scalable architecture and integrated hardware that can coherently connect numerous distant quantum nodes by exchanging multidimensional entanglement over complex-medium quantum channels is necessary for the implementation of large-scale and useful quantum networks. In [100], multichip multidimensional entanglement networks are presented, as shown in Figure 10(b). Mass-manufacturable methods were used to create quantum networking devices on silicon nanophotonic quantum circuits. Few-mode fibers (FMFs) coherently disseminated three pairs of multidimensional entangled photons that were created on a server chip across three quantum node chips. It overcame the nonunitary mode scrambling and entanglement deterioration by devising a quantum entanglement retrieval (QER) technique, which effectively recovered multidimensional entangled states dispersed throughout the multichip networks.

## 5 Dispersion control

Dispersion controllers are crucial components in the programmable applications of optical signal processors. It is increasingly in demand for the broad bandwidth, extensive group delay, and significant dispersion tuning capabilities [101], [102], [103]. Precise dispersion management enables the implementation of optical true time delaylines across a wide frequency range, ensuring proper functionality for applications like phased array antennas and beamforming [104], [105]. In addition, dispersive-propagation-based arbitrary waveform generation is vital for techniques such as pulse shaping and frequency-to-time mapping [106], [107].

**Figure 11:**
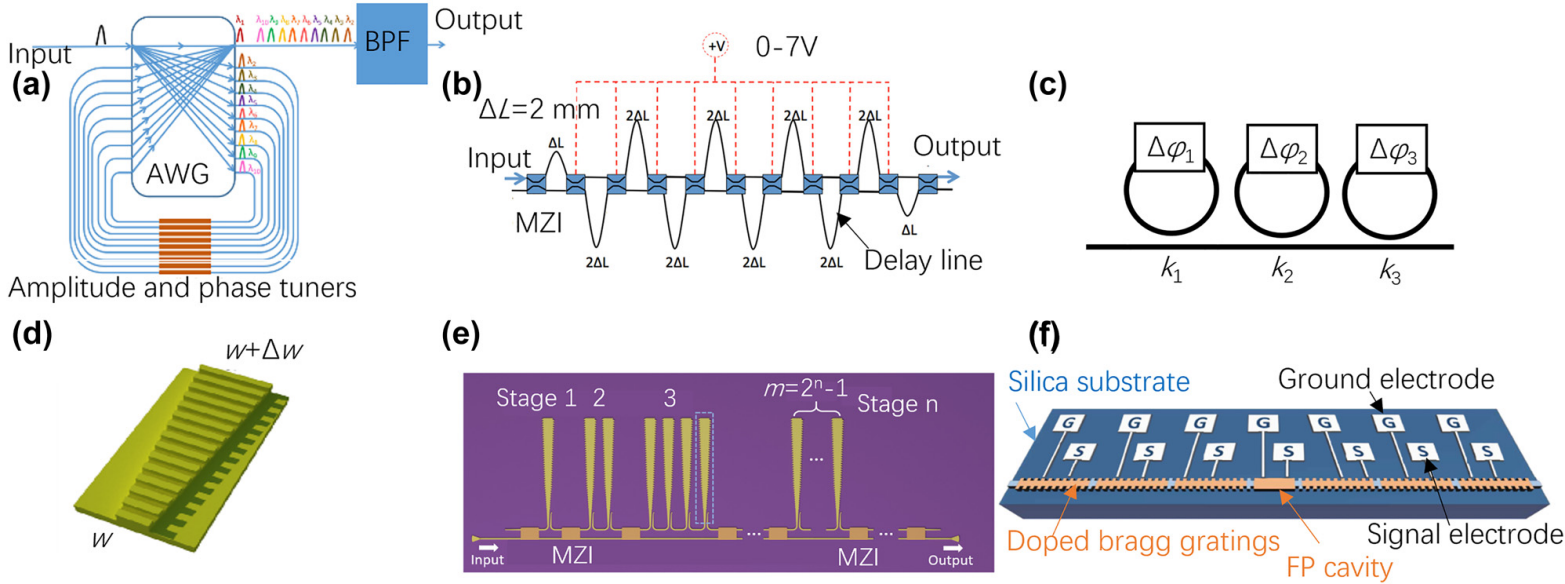
Various structures of on-chip DC. (a) Delay lines assisted with AWG [112]; (b) MZIs [110]; (c) MRRs; (d) chirped Bragg grating [111]; (e) digitally tunable dispersion controller [26]; (f) schematic view of a programmable microwave signal processor [125]. (a) Reproduced with permission [112]. Copyright 2020, IEEE. (b) Reproduced with permission [110]. Copyright 2016, Optica Publishing Group. (d) Reproduced with permission [111]. Copyright 2012, Optica Publishing Group. (e) Reproduced with permission [26]. Copyright 2023, Optica Publishing Group. (f) Reproduced with permission [125]. Copyright 2018, Nature Publishing Group.

In recent years, diverse on-chip dispersion controllers have emerged, primarily designed through the utilization of optical delaylines, MRRs [108], MZIs [109], [110], and waveguide Bragg gratings [111], providing the modulation of wavelength-dependent group delays, as outlined in Table 3. Typically, distinct group delays are generated by traversing different optical paths in the case of optical delaylines [19]. However, these delays are discrete, and the chip footprint expands with the increasing number of channels. It’s important to note that multi-channel delaylines necessitate integration with wavelength division multiplexing (WDM) devices like AWG [112], as shown in Figure 11(a). To address these challenges, an alternative method for dispersion control is managing group velocity. Cascaded MZIs can function as continuous dispersion controllers by adjusting the lengths of their in-between phase shifters, allowing for wavelength multiplexing and true time delay simultaneously [109], as shown in Figure 11(b). Nevertheless, they are hindered by large footprints and extremely narrow bandwidths. Besides, MRR-based dispersion controllers offer a compact footprint, their bandwidth is limited to less than 0.5 nm [113], [114], as shown in Figure 11(c).

**Table 3:** Summary of reported on-chip DCs.

Structure	Platform	Circulator-free	Dispersion tunable	Delay range [ps]	Bandwidth [nm]	Loss [dB/ns]	Dispersion [ps/nm]	Footprint [mm^2^]
Contra-DC [104]	Silicon	√	–	400	12	25.4	33	∼6.86 × 0.57
Spiral Bragg [118]	Silicon	–	–	628	22.5	6	−27.7	0.3 × 0.3
Spiral Bragg [119]	Silicon nitride	–	–	1440	9.2	1.875	−156.5	2.8 × 2.8
Multimode spiral Bragg [120]	Silicon nitride	√	–	2864	23	1.57	158	2 × 2
MRRs [70]	Silicon nitride	√	√	560	0.064	–	–	5.5 × 16
MZIs [110]	Silicon nitride	√	√	–	0.8	–	−550 to 550	9.89 × 22.5
Cascaded CMWG [26]	Silicon	√	√	760	20	10.53	0 to 42.8	2.4 × 0.38

Furthermore, precise thermal tuning is necessary for both MZIs and MRRs to effectively manage dispersion. In the pursuit of wide-range dispersion management with continuous wavelengths, contra-directional couplers (contra-DCs) and chirped Bragg gratings have been introduced by modulating their gradually-varied refractive-index profiles, resulting in continuous wavelength-dependent delay variations [111], [115], as shown in Figure 11(d). However, contra-DC has a complex structure due to the presence of two strip tapered waveguides with a shallow slot and grating corrugations between them. Among these options, chirped multimode waveguide gratings (MWGs) assisted with low-loss mode (de)multiplexers offer an attractive design due to their circulator-free characteristics and structural simplicity, fabrication ease, and integration compatibility [116]. Significant group delay and dispersion production rely on long-length gratings, and structures like spiral gratings have been developed to achieve substantial dispersion within compact footprints [117], [118], [119]. Additionally, cascaded MWGs provide a feasible solution to minimize space occupation, as the dispersion multiplies with an increased cascaded amount [120].

Moreover, dispersion tuning is in highly demand in various fields, including beamforming requiring dynamic delay paths [121], and microwave waveform generation with multiple frequencies [122]. A digitally-tunable dispersion controller was proposed and demonstrated in [26] for the first time by using chirped MWGs, providing high flexibility for tuning the dispersion range with a fine step conveniently, as shown in Figure 11(e). By integrating *N* stages of chirped MWGs and (*N* + 1) MZSs, the total dispersion can be freely tuned from 0 to (2^*N*^ − 1)/*D*_0_ with a step of *D*_0_, where *D*_0_ is the dispersion provided by a single chirped MWG unit. Furthermore, a dispersion controller with bilateral tuning and a larger tuning range is imperative to meet the demands of modern applications [113].

Currently, fixed multichannel delaylines is used as a common method of generating microwave photonic filters [10]. A tuning dispersion controller can help change the time delay between each tap so that the filter can be reconstructed and tuned. For arbitrary waveform generation, one can control the frequency of the generated signal by adjusting the different dispersion amount [123], [124]. As shown in Figure 11(f), a fully reconfigurable waveguide Bragg grating was proposed for programmable photonic signal processing [125]. By using the free-carrier plasma dispersion effect on silicon, the Bragg grating can be fast and electrically reconfigured, so that it can be used for high-speed programmable photonic signal processing. By controlling all bias voltages, the entire index modulation profile of the grating can be electrically reconfigured and signal processing functions including temporal differentiation, true time delay, and microwave frequency identification have been demonstrated.

## 6 Conclusions

Silicon-based on-chip signal processors have opened a path for the new generation of signal processing, thanks to its large bandwidth, low latency, programmability, efficient power consumption, and information security. As the fabrication techniques developed, large-scale silicon photonic signal processor for more powerful and flexible information processing becomes possible. In this paper, we have given a review of the recent progresses in large-scale programmable silicon photonic signal processing chips and their applications in microwave photonics, optical communications, optical computing, and quantum photonics. Table 4 shows the performance comparison of representative large-scale silicon photonic signal processors in various applications, realized with straight waveguides [21], MZSes [18], [21], MRRs [10], [25], AWGs [96], and Bragg-gratings [26], [117] by using the TO tuning mechanism. In this case, silicon optical signal processors can be tuned easily in principle by heating the phase-shifting section [47]. It is also possible to utilize the mechanisms of the EO effect or the stress-optical tuning to further lower the power consumption and improve the tuning speed [48], [49]. Among them, MZSes are still suitable for a broadband operation to simultaneously switch multiple paths as desired in various systems [73], [74], [75], [76], [77], [78], [79], [80], [81]. When constructing MZSes into mesh topologies for realizing large-scale signal processing, the random phase errors in MZI arms make the characterization and management very difficult and complicated. It is very important to minimize the random phase errors with improved structural designs and fabrication precisions. Broadened phase shifters can help to relieve the random phase errors, resulting in quasi-calibration-free configuration of optical signal processors and low power consumption for phase-error compensation [24], [75]. In contrast, an MRR switch is wavelength-selective [10], [52], [53], [54], [55], [56], and can switch any given wavelength-channels by shifting the resonance wavelength flexibly. For this case, the 3-dB bandwidth should be small when a high ER is desired. As a result, one should control the temperature critically for accurate wavelength alignments. For the case with multiple wavelength-channels, AWGs provide an attractive option because of the compact size and the scalable input/output ports. Currently, the channel number of AWG is now extended to 32 for reservoir computing [96].

**Table 4:** Comparison of the reported large-scale silicon optical signal processors.

Year	Size	Structure	Waveguide loss (dB/cm)	Total loss (dB)	Power efficiency (mW/π)	Tuning mechanism	Configuring methods	Total power consumption (mW)	Functions
2019 [80]	240 × 240	Vertical adiabatic coupler	0.45	9.8	0.042	MEMS	Manual	20.2	MEMS switch array
2020 [73]	8 × 8	MZIs	0.8	6.6	–	TO		1500	Optical switch array
2023 [74]	32 × 32	MZI	0.58 dB/MZI	23–28	–	TO	GD		Thermo-optic switch array
2016 [76]	16 × 16	MZI	–	14	3.28–5.88	EO	PSO	1170	–
2017 [77]	32 × 32	MZI	0.53	12.9–18.5	–	EO	–	247.4/542.3	Electro-optic switch array
2018 [48]	64 × 64	MZI	–	30.7–48.3	–	TO	–	–	Thermo-optic silicon photonic switch
2019 [78]	5 × 5	MZI	2	8	300–360	TO	–	3300	–
2023 [79]	32 × 32	MZI	1	12.88	30.55	TO	–	980	–
2023 [81]	16 × 16	Split waveguide crossing	8.2	10.9	0.54 pJ (EC)	MEMS	Manual	–	MEMS switch array
2017 [9]	4 × 4	MZIs	0.3	3	–	TO	SGD	–	Speech recognition
2020 [20]	8 × 8	MZIs	–	–	–	TO	GD	–	Tunable filter/switch array/descrambler
2021 [89]	8 × 8	MZIs	–	–	–	TO	BP/GD	–	Logic gates/handwriting recognition
2023 [75]	16 × 16	MZI	–	4–8	23	TO	Calibration-free	182 mW	Thermo-optic switch array
2007 [66]	8 × 1	Microrings	0.55	12	250	TO	–	8000	Photonic beamformer
2020 [21]	8 × 1	Delaylines and PD	1.3	7	20	TO	–	1450	Photonic beamformer
2023 [24]	4 × 1	Delaylines and PD	0.28	3–3.4	22	TO	Calibration-free	540	Beamformer/Tunable filter/arbitrary waveform generation
2023 [96]	32 × 32	AWG	–	–	–	TO	PSO		Predicting Macky-Glass series

At present, numerous programmable silicon photonic signal processors have been successfully demonstrated various applications, however, most of them have a limited scale (≤16 × 16 [9], [20], [89]), mainly due to the limitation of the excess loss, the robustness, the power consumption and the configuring complexity. With a larger-scale configuration, the advantage in total energy efficiency and latency for signal processing is expected to be further enhanced [27]. Considering the entire signal processing systems including photonic, optoelectronic, and electronic devices/circuits, some empirical evaluation results indicate that silicon optoelectronic signal processor will possibly outperform digital logic circuits in terms of the energy efficiency and the speed when the matrix configuration exceeds 128 × 128 [126]. Although some devices have been extended to the scale of 256 × 256 [91] and 240 × 240 (MEMS) [80], the total loss is still high for their practical applications and more efforts should be made in the future. To tackle those problems, the broadened-waveguide techniques have been proposed to relief the pressures from the propagation loss and high uniformity in the fabrication [30], [31]. To be more specifically, the scattering loss introduced by the rough sidewalls and the accumulated phase errors can be minimized as the core width is broadened to be far beyond the singlemode regime. In addition to the waveguide loss, the loss of the basic building blocks, including 3-dB couplers, MZIs, ring resonators and other active components would accumulate to increase the total system loss, which prevents the practical applications of the on-chip programmable signal processors. The most straightforward method to eliminate this problem is to optimize the excess loss of all the components as well as the coupling loss. Secondly, integrating more components on a single chip is also helpful for low-loss and stable operation [58], [127]. Lastly, employ high responsivity and high gain-bandwidth-product photodetector to detect low-power optical signals with high sensitivity [8].

In addition to the techniques shown in the papers above, there are also some other key aspects for the realization of the large-scale optical signal processors, such as advanced packaging/assembling, circuits (ICs) for automatic management/control. Currently, manufactures have offered high-performance large-scale photonic devices implemented with in-house package/assembly processes and multi-disciplinary design/testing. For high-efficiency electrical controlling of large-scale signal processors with many heaters to be driven simultaneously, the approach of improved digital pulse width modulation (PWM) together with the optimized heating structures have been proposed to eliminates the need for DACs, minimize optical power penalties, and reduce the power consumption [128].

Finally, although silicon photonics are able to support both active and passive components for optical signal processing with high integration density, silicon is not a direct bandgap material thus no light can be generated. On the other hand, laser is an important component for signal processing, which cannot be absent. Fortunately, multiple strategies have been explored to integrate III–V lasers tightly with silicon photonic integrated circuits [129], including hybrid integration [130], wafer-bonding-based heterogeneous integration [131], and monolithic integration based on direct epitaxial growth.

As a summary, silicon photonics has shown great potential for realizing large-scale photonic integrated circuits, including the optical signal processors consider here. It is expected to further scale the photonic integrated chip if the reliability and uniformity of the fundamental elements are improved greatly with smart designs and advanced fabrication technologies in the future.
